# Seroprevalence of HBV and HCV in blood donors: A study from regional blood transfusion services of Nepal

**DOI:** 10.4103/0973-6247.67026

**Published:** 2010-07

**Authors:** B. R. Tiwari, P. Ghimire, S. R. Kandel, M. Rajkarnikar

**Affiliations:** *Department of Quality Assurance, Nepal Red Cross Society, Central Blood Transfusion Service, Kathmandu, Nepal*; 1 *Central Department of Microbiology, Tribhuvan University, Kathmandu, Nepal*

**Keywords:** Blood donors, hepatitis B virus, hepatitis C virus, Nepal, seroprevalence

## Abstract

**Background and Objective:**

Hepatitis B and hepatitis C are significant health problems that might involve the late sequel of liver cirrhosis and hepatocellular carcinoma. A high prevalence of hepatitis B virus (HBV) and hepatitis C virus (HCV) in blood donors poses an increased risk of window period transmission through blood transfusion. The present study aimed to know the seroprevalence of hepatitis B virus (HBV) and hepatitis C virus (HCV) among blood donors in regional blood transfusion services of Nepal.

**Materials and Methods:**

This was a retrospective study conducted among blood donors in Banke (5,211), Morang (5,351), and Kaski (5,995) blood transfusion services. Serum samples were tested for hepatitis B surface antigen (HBsAg) and anti-HCV antibodies using rapid enzyme immunoassays. The donors information was collected via the donor record register through their respective blood transfusion services. The software “Winpepi ver 3.8” was used for statistical analysis.

**Results:**

The seroprevalence rate of HBV was highest in the Banke (1.2%) followed by Biratnagar (0.87%) and Kaski (0.35%) (*P* < 0.0001). The seroprevalence of HCV was highest in the Morang (0.26%) followed by Kaski (0.16%) and Banke (0.11%) (*P* > 0.05). The seroprevalence of HBV was significantly higher than HCV in all three blood transfusion services. The burden of HBV as well as HCV seems to be higher in male donors (*P* > 0.05).

**Conclusion:**

The study revealed that the seroprevalence of HBV was alarmingly higher in two of the three blood transfusion services. Implementation of community-based preventive measures and improved strategies for safe blood supply might prove useful to decrease the seroprevalence.

## Introduction

Hepatitis B virus (HBV) infection is one of the most common infectious diseases in the world with significant acute and chronic morbidity and thus has become a global public health problem. The presence of HBsAg in serum indicates active HBV infection, either acute or chronic. Usually, HBsAg is the first serologic marker in acute HBV infection and is detected 2–4 weeks before the alanine aminotransferase (ALT) level becomes abnormal and 3–5 weeks before symptoms or Jaundice.[1] World Health Organization (WHO) has estimated that more than 2 billion people in the world have been infected with HBV at some time in their lives and about 350 million people worldwide are HBV carriers with the majority in developing world mainly in Asia and Africa.[2] In Nepal, seroprevalence of HBsAg has been reported ranging from 0.3% to 4.0% in general population by various studies conducted from 1990 to 2003.[3–9] Among Nepalese blood donors, HBsAg seroprevalence has been reported ranging from 0.88% to 1.26%.[10–12]

Hepatitis C virus (HCV) continues to be a major disease burden in the world. In 1997, WHO estimated a worldwide prevalence of about 3% with the virus affecting 170 million people worldwide and 3 to 4 million new infections each year.[13] Among the viral hepatitis, HCV is dreadful in the aspect that its morbidity rate is high as it establishes a state of chronic infection in as many as 85% of acutely infected patients whereas about 15% of acutely infected patients spontaneously clear the infection.[14,15] In Nepal, seroprevalence of anti-HCV antibodies among general population and blood donors has been reported ranging from 0.31.7%.[16–20]

Nepal is a country in South Asia, divided administratively into five development regions viz. Eastern, Central, Western, Mid Western, and Far Western development regions. The three regional blood transfusion services of Nepal viz. Morang, Banke, and Kaski, are situated in Eastern, Western, and Mid Western development regions, respectively. As Nepal is a country with considerable diversity of people in race, ethnic group, and in its socioeconomic condition, the status of hepatitis B and C might be different in different regions. A comparative study from three geographically and socioeconomically different regions would reveal important data that can be used to make plan and policy for assuring safe blood and would reveal the burden of disease in healthy looking general population in respective regions.

## Materials and Methods

This was a retrospective cross-sectional study conducted in three regional blood transfusion services of Nepal over a period of 1 year from July 2006 to June 2007. Blood donors were selected if they fulfilled all the criteria to be eligible for donation as described by the standard operating procedure of Nepal Red Cross Society, blood transfusion service. All blood donors donating blood in respective blood transfusion services or in mobile camps were included in the study. A total of 16,557 blood donors, namely 5,351 donors in Morang, 5,211 in Banke, 5,995 in Kaski, were subjected for routine mandatory screening for HBsAg and anti HCV antibodies by an enzyme immunoassay-based rapid tests according the standard protocol described by respective company (Hepacard, J. Mitra and Co., New Delhi, India and HCV TRI-DOT, J. Mitra and Co., New Delhi, India). Initially reactive sera were confirmed by repeat testing. Before drawing the blood each donor was requested to fill a blood donor’s form. Blood samples were tested anonymously and confidentiality was maintained as per standard guidelines by Nepal Red Cross Society, blood transfusion service. The statistical analysis was done using the software “Winpepi ver 3.8” and the significance of difference in seroprevalence was tested by the Chi-square test.

## Results

In Morang (Biratnagar) Blood Transfusion Service, a total of 5,351 donors were screened in whom 84.8% (4,537/5,351) were males and 15.2% (814/5,351) were females. Among them 47 donors were found seropositive for HBV giving the seroprevalence of 0.87% (47/5,351, 95% CI= 0.6-1.2%). The HBV seroprevalence in male donors was 0.96% (44/4,537) and in female donors was 0.36% (3/814) [Table 1]. The seroprevalence of HCV was found 0.26% all of whom were males (14/5,351, 95% CI= 0.1-0.4%). The difference in seroprevalence for HBV and HCV was statistically significant (*P*< 0.0001).

**Table 1:** HBV and HCV seroprevalence in regional blood transfusion services of Nepal

Regional Blood Transfusion Service	Total donors	Males	Females	HBV seropositive	Seroprevalence (%)	HCV seropositive	Sereprevalence (%)
Morang	5,351	4,537	814	47	0.87	14	0.26
Banke	5,211	4,766	445	63	1.2	6	0.11
Kaski	5,995	5,245	750	21	0.35	10	0.16

The difference in seroprevalence of HBV observed among the three regional blood transfusion services was statistically significant (*P*< 0.0001) but for HCV, the seroprevalence was not statistically significant (*P* > 0.05).

In Banke (Nepalgunj) Blood Transfusion Service, a total of 5,211 donors were screened in whom 91.5% (4,766/5,211) were males and 8.5% were females. Among them 63 were found to be seropositive for HBV giving the seroprevalence of 1.20% (63/5,211, 95% CI= 0.9-1.5%) of whom 62 were males and only 1 was female. The seroprevalence of HCV was 0.11% (6/5211, 95% CI= 0.0-0.3%), of whom all were males [Table 1]. The difference in seroprevalence for HBV and HCV was statistically significant (*P*< 0.0001)

In Kaski (Pokhara) Blood Transfusion Service, a total of 5,995 blood donors were screened in whom 87.5% (5,245/5,995) were males and 12.5% (750/5,995) were females. Among them 21 donors were found to be seropositive for HBV giving the seroprevalence of 0.35% (21/5,995, 95% CI= 0.2-0.5%) of whom 20 were males and only a single seropositive donor was female. The seroprevalence of HCV was 0.16% (10/5,995, 95% CI= 0.1-0.3%) of whom 8 were males and 2 were females [Table 1]. The difference in seroprevalence for HBV and HCV was statistically significant (*P*< 0.05).

## Discussion

The seroprevalence of HBV was highest in Banke followed by Biratnagar and then by Kaski (*P*< 0.0001). However, a study from central blood transfusion service, Kathmandu has shown a HBV seroprevalence of 0.53%.[21] The seroprevalence of HBV observed in Banke and Biratnagar was considerably higher than reported by a study from central blood transfusion service, Kathmandu; however, the seroprevalence observed in Pokhara was relatively lower.[21] The seroprevalence of HBV from Morang and Banke Blood Transfusion Services was similar to the seroprevalence reported by other studies conducted among blood donors in Nepal, but the seroprevalence from Kaski Blood Transfusion Service was relatively lower compared to these studies.[10–12]

The seroprevalence of HCV was highest in the Morang followed by Kanski and Banke (*P*> 0.05). However, a study by Karki *et al*., has reported that the nationwide prevalence of HCV at the same time period was 0.54% and in central blood transfusion service it was 0.7%.[22] Compared to the above report, the seroprevalence of HCV in all three regional blood transfusion services was considerably lower than the seroprevalence in Kathmandu valley. Similarly, the seroprevalence from all three blood transfusion service was lower than other studies regarding seroprevalence of HCV from blood donors and/or general population published from the Nepalese population.[16–19] Similar to the present study, Shrestha *et al*., have reported a 0.35% seroprevalence of HCV among healthy adults with no significant difference in seroprevalence by geographic and development regions of Nepal.[20]

The study revealed that the problem of HBV as well as HCV was highest in Morang. The higher seroprevalence is the indicative of higher risk of transmission of blood borne infections due to serological window period. So, stringent donor selection criteria, effective donor education, community education and intervention to lower the prevalence in general population, blood donation by regular volunteer donors and donor notification, and counseling of seropositive donors might prove useful to lower the seroprevalence in blood donors. The present study showed that the seroprevalence of HBV was higher than HCV in all three blood transfusion service. So, special intervention programs should be planned to aware the general public about the highly infectious nature of HBV and its mode of transmission. Hepatitis B is vaccine preventable, so the need, suitability, and feasibility of mass vaccination programs in populations showing such higher prevalence should be studied.
